# Main predictors of periphyton species richness depend on adherence strategy and cell size

**DOI:** 10.1371/journal.pone.0181720

**Published:** 2017-07-24

**Authors:** Vanessa Majewski Algarte, Tadeu Siqueira, Victor Lemes Landeiro, Liliana Rodrigues, Claudia Costa Bonecker, Luzia Cleide Rodrigues, Natália Fernanda Santana, Sidinei Magela Thomaz, Luis Mauricio Bini

**Affiliations:** 1 Departamento de Ciências Biológicas, Programa de Pós-graduação em Ecologia de Ambientes Aquáticos Continentais, Núcleo de Pesquisas em Limnologia, Ictiologia e Aquicultura, Universidade Estadual de Maringá, Maringá, Paraná, Brazil; 2 Departamento de Ecologia, Instituto de Biociências, UNESP—Universidade Estadual Paulista, Rio Claro, São Paulo, Brazil; 3 Departamento de Botânica e Ecologia, Instituto de Biociências, Universidade Federal do Mato Grosso, Cuiabá, Mato Grosso, Brazil; 4 Departamento de Ecologia, Universidade Federal de Goiás, Goiânia, Goiás, Brazil; VIT University, INDIA

## Abstract

Periphytic algae are important components of aquatic ecosystems. However, the factors driving periphyton species richness variation remain largely unexplored. Here, we used data from a subtropical floodplain (Upper Paraná River floodplain, Brazil) to quantify the influence of environmental variables (total suspended matter, temperature, conductivity, nutrient concentrations, hydrology, phytoplankton biomass, phytoplankton species richness, aquatic macrophyte species richness and zooplankton density) on overall periphytic algal species richness and on the richness of different algal groups defined by morphological traits (cell size and adherence strategy). We expected that the coefficients of determination of the models estimated for different trait-based groups would be higher than the model coefficient of determination of the entire algal community. We also expected that the relative importance of explanatory variables in predicting species richness would differ among algal groups. The coefficient of determination for the model used to predict overall periphytic algal species richness was higher than the ones obtained for models used to predict the species richness of the different groups. Thus, our first prediction was not supported. Species richness of aquatic macrophytes was the main predictor of periphyton species richness of the entire community and a significant predictor of the species richness of small mobile, large mobile and small-loosely attached algae. Abiotic variables, phytoplankton species richness, chlorophyll-a concentration, and hydrology were also significant predictors, depending on the group. These results suggest that habitat heterogeneity (as proxied by aquatic macrophytes richness) is important for maintaining periphyton species richness in floodplain environments. However, other factors played a role, suggesting that the analysis of species richness of different trait-based groups unveils relationships that were not detectable when the entire community was analysed together.

## Introduction

Species richness is the simplest and oldest measure of biodiversity [1]. Considering the theoretical and practical importance of this measure, a major challenge in community ecology is to quantify and understand how richness varies in space and time [2, 3]. In freshwater ecosystems, periphyton species richness–mainly consisting of algae–is usually linked to the influence of local abiotic and biotic factors such as nutrient levels, light, grazing [4, 5], and substrate characteristics [6]. An increase in nutrient concentrations is, in general, positively related to periphyton species richness, whereas herbivores may reduce richness due to the removal of rare species from the periphytic matrix [7, 8]. Recent studies have also shown that habitat heterogeneity and regional microalgae species richness have a strong influence on richness of periphyton algae communities [9–11]. Finally, hydrological factors may be as important as nutrient levels in determining periphyton growth [12, 13].

The explanatory power of models predicting periphyton species richness varies conspicuously, although it tends to be low (see [9, 14, 15]). In a study carried out by Passy [9], for example, a model allowing for variables related to stream and drainage basin conditions explained 11–12% of the variation in species richness of diatoms in environments with different hydrological regimes in the USA. This contrasts with the findings by recent studies showing that the explanatory power of species richness models, in particular, and of ecological models, in general, ranged between 50 and 60% [16, 17]. The low capacity to predict algal species richness in freshwater systems may be due to, for example, a lack of key variables and/or sampling along short environmental gradients [18]. Moreover, macroecological studies suggest that species richness, as an aggregate variable consisting of the sum of the presence of different species with different environmental requirements, is hard to predict and exhibits a high degree of “noise” [19, 20]; but see [21]. Therefore, using the deconstructive approach suggested by Marquet et al. [20] and grouping species according to shared biological traits may be an alternative to increase the predictive power of the models as well as to help understand ecological patterns. The predictive ability of species richness models is expected to increase when different groups are modelled separately, using the deconstruction principle, because species within a trait group are assumed to respond similarly to environmental gradients.

Periphytic algae vary in size, morphology, and form of attachment, enabling the classification of the species into groups [22]. High morphological diversity among species, for example, may be observed in periphytic algal communities [23]. This diversity extends from coenobial algae that exhibit flagella, processes, lobes and mucilaginous sheaths to algae ranging from small-to large-sized and deprived of such traits [22]. The processes, lobes, and mucilage are cell shape adaptations that provide longer suspension time for algae in the water column [24]. Thus, algae with the aforementioned traits may be more likely to disperse passively once removed from the substrate when compared with algae without such traits. Similarly, small algae are also more likely to be carried by water flow than large algae [25]. The results of biological trait-based analysis have, in general, enabled a more comprehensive understanding of the structure and dynamics of periphytic algae communities (see [15, 26, 27]).

In this study, we grouped periphytic algae of a subtropical floodplain in Brazil according to size and attachment strategies. We then modelled species richness of the different trait groups as a function of abiotic (temperature, conductivity, nutrient concentrations, hydrology and total suspended matter) and biotic variables (phytoplankton species richness, macrophyte species richness, zooplankton density and chlorophyll-*a* concentration in the water column). We tested the hypothesis that the relative importance of local abiotic and biotic factors in predicting species richness would differ among the whole algal community and the groups formed according to traits. First, we expected that the fitted models for species richness of the different trait-based groups would be better adjusted than the fitted model for species richness of the whole periphytic algae community. Second, we explored the role of explanatory variables that are seldom considered in models of algal species richness (i.e., proxies for algal species pool, environmental heterogeneity and grazing pressure). We predicted that periphyton species richness would correlate positively with environmental heterogeneity and size of the species pool of potential colonists. Grazing pressure was predicted to correlate negatively with periphyton species richness.

## Materials and methods

License for sampling was provided by "Sistema de Autorização e Informação em Biodiversidade" (SISBIO-ICMBio), number 22442–1.

### Study area

The Upper Paraná River basin drains an area corresponding to ca. 10.5% of the total area of Brazil. In a stretch located between the states of Mato Grosso do Sul and Paraná (22°40’-22°50’S and 53°10’-53°24’W) occurs a 230-km-long floodplain that represents the last dam-free stretch of the Paraná River in Brazil [28]. The Baía and Ivinhema rivers are two important right-bank tributaries of the Paraná River, and together, they constitute three major sub-basins in this floodplain [29, 30]. These sub-basins are distinct from each other and have high variability in abiotic factors (e.g., water transparency, nutrient content and depth) and in their aquatic communities [28, 29].

The study was carried out in a 60-km-long stretch of the Upper Paraná River floodplain. We sampled the communities and explanatory variables simultaneously in 30 sites in March 2010 (S1 Table), which corresponded to a high water period. The water level during the study period ranged from 3.26 m to 5.07 m.

### Sampling and formation of periphytic algal trait groups

We collected periphytic algae from petioles of *Eichhornia azurea* (Sw.) Kunth. In each site, we sampled two petioles of *E*. *azurea* from different plants (i.e. the total number of petioles was 60) at each sampling site. This species was chosen because it is one of the most widespread in the Upper Paraná River floodplain, allowing for comparisons among sites and controlling for differences in the periphytic communities that would otherwise be observed if the samples were taken from different species of aquatic macrophytes. We chose petioles that were visually in the same stage of development (i.e. avoiding too young and too old petioles) in an attempt to control the colonization time of the attached algae. For instance, very young petioles or those with signs of senescence were not sampled. The petioles were placed in separated Wheaton bottles (150 ml) and transported to the laboratory in Styrofoam box containing ice. The periphytic material was removed by gently scraping the surface of 60 petioles (mean area: 34.12 cm^2^ ± 8.7) with the aid of a stainless-steel blade and jets of distilled water. The scraped material was preserved with Lugol’s solution [31]. The samples were analyzed in an inverted microscope (at 400x) according to methods described in Utermöhl [32] and Lund et al. [33]. Each sample was analyzed until the stabilization of the species accumulation curve. Algal cells were identified to the lowest possible taxonomic level (usually species) according to the classical literature (see [34] for a detailed list of references). The number of taxa recorded in each site was regarded as the species richness.

We classified algae species into six trait groups according to their size and attachment ability (S2 Table): small/mobile (≤ 70 μm), large/mobile (>70 μm), small/loosely attached (≤ 70 μm), large/loosely attached (>70 μm), small/firmly attached (≤ 70 μm), large/firmly attached (>70 μm). We used the species richness data for the whole community and for each of these groups separately as response variables (see below).

### Limnological and hydrological variables

We filtered water samples through Whatman GF/F filters under low pressure (< 0.5 atm) to quantify the chlorophyll-*a* content of the water (μg L^-1^). Then, the filters were kept frozen until chlorophyll-*a* extraction, and pigment concentrations were determined by spectrophotometry [35]. Chlorophyll-*a* content is a measure of phytoplankton biomass [36] and may be a rough indicator of productivity [37]. Water temperature and conductivity were measured *in situ* using field meters. We also measured total suspended matter (TSM), according to the gravimetric method [36], and nutrient concentrations (total phosphorus and inorganic nitrogen), according to the methods described by Mackereth et al. [38], Giné et al. [39] and Koroleff [40]. Water samples were taken at subsurface depth (ca. 30 cm) from each sampling site. We created a dummy variable (factor with two levels) to represent hydrology and contrast lotic (0.0) and lentic (1.0) sampling sites. Lotic sampling sites were localized in low-flow, cut-off channels.

### Planktonic and aquatic macrophyte communities

The number of algal species from planktonic samples may be a proxy of the species pool size that could potentially colonise the periphyton [41]. For instance, using data obtained in a long-term ecological research program, from 2000 to 2008, (http://www.peld.uem.br/Relat2008/index08.htm), we found that phytoplankton and periphytic samples shares a large number of genera (ca. 40.1% of 222 genera). Thus, we sampled the phytoplankton community at the subsurface of the water column (20 cm depth) in the pelagic region of the study sites. Samples were collected directly with 150-mL Wheaton flasks, fixed with 5% acetic Lugol's solution and stored in the dark [31]. Samples were analysed in an inverted microscope equipped with a 40x objective and the number of taxa recorded in each sample was regarded as the species richness. We note that the dam upstream of our study area most likely impacted the phytoplankton community, changing the regional species pool. Even so this is the species pool available to colonize all environments downstream (in addition to the one from the own floodplain).

Because periphytic algae may potentially be an important food source for zooplankton in shallow environments [42] we used the density of herbivorous zooplankton as a proxy for herbivory. The zooplankton community was sampled in the subsurface of the pelagic region of the sites by filtering 1000 L of water with the aid of a water pump through a 68-μm plankton net. The collected material was placed in labelled polyethylene flasks and fixed in a 4% formaldehyde solution buffered with calcium carbonate. Zooplankton samples were analysed (counting and identification) using a Sedgewick-Rafter chamber in an optical microscope. The organisms were quantified by counting three 2.5-mL sub-samples obtained with a Hensen-Stempel pipette. A minimum of 80 individuals was counted in each sub-sample [43]. The total density of zooplankton species that potentially consume periphytic algae (according to [44]) was expressed in terms of individuals per cubic meter (ind.m^-3^). Species were identified according to the references listed in S1 File. We believe that our proxy for grazing is defensible because of two reasons: (i) the petioles of *Eichhornia azurea*, from where the periphytic samples were taken, develop few centimeters below the water surface and (ii) the petioles grow towards the pelagic region of lakes and canals, where zooplankton are usually abundant. We recognize that phytophilous invertebrates would also be an alternative proxy. However, data on these organisms lacked in our samplings.

Aquatic macrophytes with different morphological structures lead to a more heterogeneous microhabitat for the periphytic community, and aquatic macrophyte richness may be an indicator of environmental heterogeneity [45]. Thus, during 10 minutes, presence and absence data on aquatic macrophytes were recorded from a boat moving at a slow and constant speed along the shoreline regions of the sampling sites. Submerged plants were collected using a rake in each sampling site. The fact that we always used the same substrate (*E*. *azurea* petioles) does not influence the idea of using the richness of aquatic macrophytes as a proxy for environmental heterogeneity. Instead, we believe that the use of only *E*. *azurea* as substrate was pivotal to control for other confounding effects. Our reasoning was that an increase in aquatic macrophytes species richness, associated with different algal species from the periphytic community, would increase the pool of potential colonizers of the periphytic community associated to *E*. *azurea*. Also, the richness of plants is a general proxy of environmental heterogeneity in species richness research [45]. One can argue about the possible allelopathic effect of *E*. *azurea* on periphytic species richness. However, this effect can be ruled out because a previous study indicated a high species composition similarity between an artificial substrate and *E*. *azurea* [46].

### Data analysis

We used a Principal Component Analysis (PCA) to summarize the limnological variables (water temperature, conductivity, TSM, total phosphorus and inorganic nitrogen). The first principal component explained (PC1) most of the variance in the data (58.17%) and was retained for further analysis as it was the only one with an eigenvalue higher than 1.0 (Kaiser-Guttman criterion; [47]). PC1 contrasted sites with high temperature and total phosphorus concentration (localized in the Ivinhema e Baía sub-basins) from sites with high ionic, TSM and inorganic nitrogen concentrations (localized in the Paraná sub-basin; see Results section).

PC1, hydrology, chlorophyll-*a* concentration, phytoplankton species richness, herbivorous zooplankton density, and macrophyte species richness were the explanatory variables in our models for predicting periphytic species richness. The response variables were the total species richness of the periphytic community and the richness of each individual trait group (*n* = 30 sites). For each response variable, we estimated six Generalized Least Square (GLS) models with different spatial correlation structure following Zuur et al. [48]: (i) no spatial structure in the residuals, (ii) exponential correlation, (iii) Gaussian correlation, (iv) linear correlation, (v) rational quadratic correlation, and (vi) spherical correlation. We selected the model with the lowest Akaike Information Criterion (AIC) value [48]. Despite the use of GLS models with different spatial structures, we cannot rule out the effects of complex water flow dynamics and passive dispersal in determining spatial variation in species richness of periphytic algae. For comparative purposes, we calculated the coefficients of determination according to ordinary least-squares (OLS) models given the problems in estimating this quantity for GLS models [49].

The statistical analyses were carried out using R software version 2.15 [50]. The PCA was carried out using the package *vegan* [51], while the GLS models were estimated using the package *nlme* [52].

## Results

We identified 392 taxa of periphytic algae distributed among 122 genera. Highest species richness was found for small/loosely attached algae, followed by large/loosely attached, small/firmly attached, small/mobile, large/firmly attached and large/mobile (Table 1). The explanatory variables (i.e., data on abiotic variables, chlorophyll-*a* concentration, phytoplankton species richness, zooplankton density, and macrophyte species richness) varied widely among the sites. The highest coefficients of variation were recorded for inorganic nitrogen, zooplankton density, TSM and chlorophyll-*a* concentrations (Table 2).

**Table 1:** Species richness variation of periphytic algae from the Upper Paraná River floodplain and of the groups formed according to morphological traits (*n* = 30).

Groups	Minimum	Maximum
Whole Community	51	158
Small/mobile	4	19
Large/mobile	0	6
Small/Loosely attached	12	79
Large/Loosely attached	5	37
Small/Firmly attached	15	31
Large/Firmly attached	7	16

**Table 2:** Minimum (Min), maximum (Max), mean values and coefficients of variation of the limnological variables and of the phytoplankton, macrophyte and zooplankton datasets in environments of the Upper Paraná River floodplain (*n* = 30). The first five variables in this Table were summarized by a Principal Component Analysis (see Results section).

	Min	Max	Mean	CV (%)
Temperature (°C)	27.4	31.3	28.75	2.68
Conductivity (μS cm^-1^)	24	70.2	46.5	31.33
Total Suspended Matter (mg L^-1^)	0.22	7.25	1.14	125.86
Inorganic nitrogen (μg L^-1^)	0.05	360.4	68.9	150.38
Total phosphorus P (μg L^-1^)	13.9	68.43	35.48	35.09
Chlorophyll-*a* (μg L^-1^)	0.8	23.58	5.75	105.6
Phytoplankton (richness)	3	59	27.3	51.52
Macrophyte (richness)	10	34	22.1	31.47
Zooplankton (ind.m^-3^)	748	136,890.3	20,675.8	147.39

In what follows, the results of best models, which showed different autocorrelation structures, are presented (S2 File). Total species richness was positively correlated with macrophyte species richness and negatively correlated with PC1 (adjusted *R*^2^ = 0.60; Table 3). Thus, total species richness increased with total phosphorus concentration and temperature (both negatively correlated with PC1: loadings = -0.72 and -0.63, respectively) and decreased with conductivity, TSM and inorganic nitrogen concentration (variables positively correlated with PC1: loadings = 0.73, 0.86, 0.84, respectively). Small/mobile species richness was positively correlated with macrophyte species richness, phytoplankton species richness and chlorophyll-*a* concentration. The partial regression coefficient associated with zooplankton density was nearly significant (adjusted *R*^2^ = 0.57; Table 4). Large/mobile species richness was also positively correlated with macrophyte species richness and with the dummy variable representing hydrology, indicating higher species richness in lentic sites than in lotic sites (adjusted *R*^2^ = 0.21; Table 4). Variations in species richness of small (adjusted *R*^2^ = 0.44) and large loosely (adjusted *R*^2^ = 0.59) attached algae were significantly accounted for by macrophyte species richness and by PC1, respectively. For large/loosely attached algae, the partial regression coefficient associated to aquatic macrophyte species richness was nearly significant (Table 5). We did not detect significant relationships between our explanatory variables and small/firmly attached algae species richness (adjusted *R*^2^ = 0.21). However, we detected a significant positive relationship between the species richness of large/firmly attached algae and phytoplankton species richness (adjusted *R*^2^ = 0.23; Table 6).

**Table 3:** Intercept, partial regression coefficients (± SE) and associated *t*-tests for periphytic species richness regressed against our explanatory variables in the Upper Paraná River floodplain. Significant results are highlighted in bold.

	Estimate	*SE*	*t*	*P*
Intercept	4.050	0.331	12.24	0.0000
Chlorophyll-*a*	0.117	0.072	1.63	0.1172
Zooplankton density	-0.042	0.041	-1.02	0.3187
Phytoplankton richness	0.007	0.004	1.77	0.0894
**Macrophyte richness**	**0.014**	**0.006**	**2.39**	**0.0255**
**Environment (PCA-Axis 1)**	**-0.279**	**0.117**	**-2.38**	**0.0261**
Hydrology	0.165	0.106	1.56	0.1333

**Table 4:** Intercept, partial regression coefficients (± SE) and associated *t*-tests for the species richness of small and large mobile algae regressed against our explanatory variables in the Upper Paraná River floodplain. Significant results are highlighted in bold.

	Estimate	*SE*	*t*	*P*
Small/mobile				
Intercept	2.007	0.342	5.86	0.0000
**Chlorophyll-*a***	**0.184**	**0.063**	**2.93**	**0.0075**
Zooplankton density	-0.071	0.036	-1.97	0.0605
**Phytoplankton richness**	**0.009**	**0.003**	**2.67**	**0.0137**
**Macrophyte richness**	**0.015**	**0.005**	**2.92**	**0.0077**
Environment (PCA-Axis 1)	-0.147	0.113	-1.30	0.2057
Hydrology	0.057	0.090	0.64	0.5307
Large/mobile				
Intercept	0.382	0.923	0.41	0.6829
Chlorophyll-*a*	0.255	0.143	1.78	0.0880
Zooplankton density	-0.129	0.082	-1.57	0.1309
Phytoplankton richness	0.004	0.008	0.55	0.5892
**Macrophyte richness**	**0.033**	**0.012**	**2.84**	**0.0093**
Environment (PCA-Axis 1)	-0.014	0.261	-0.05	0.9581
**Hydrology**	**0.456**	**0.204**	**2.24**	**0.0349**

**Table 5:** Intercept, partial regression coefficients (± SE) and associated *t*-tests for the species richness of small and large loosely attached algae regressed against our explanatory variables in the Upper Paraná River floodplain. Significant results are highlighted in bold.

	Estimate	*SE*	*t*	*P*
Small/loosely				
Intercept	2.410	0.579	4.16	0.0004
Chlorophyll-*a*	0.107	0.126	0.85	0.4046
Zooplankton density	-0.011	0.072	-0.16	0.8762
Phytoplankton richness	0.012	0.007	1.71	0.0998
**Macrophyte richness**	**0.022**	**0.010**	**2.13**	**0.0438**
Environment (PCA-Axis 1)	-0.298	0.205	-1.45	0.1599
Hydrology	0.165	0.185	0.89	0.3819
Large/loosely				
Intercept	2.423	0.523	4.63	0.0001
Chlorophyll-*a*	0.068	0.114	0.59	0.5589
Zooplankton density	-0.058	0.065	-0.89	0.3816
Phytoplankton richness	0.004	0.006	0.58	0.5656
Macrophyte richness	0.019	0.009	2.01	0.0557
**Environment (PCA-Axis 1)**	**-0.668**	**0.186**	**-3.60**	**0.0015**
Hydrology	0.332	0.168	1.98	0.0600

**Table 6:** Intercept, partial regression coefficients (± SE)and associated *t*-tests for the species richness of small and large firmly attached algae regressed against our explanatory variables in the Upper Paraná River floodplain. Significant results are highlighted in bold.

	Estimate	*SE*	*t*	*P*
Small/firmly				
Intercept	2.906	0.225	12.89	0.0000
Chlorophyll-*a*	0.036	0.049	0.73	0.4724
Zooplankton density	-0.010	0.028	-0.36	0.7257
Phytoplankton richness	0.002	0.003	0.72	0.4772
Macrophyte richness	0.004	0.004	0.96	0.3461
Environment (PCA-Axis 1)	-0.082	0.080	-1.03	0.3151
Hydrology	0.129	0.072	1.79	0.0864
Large/firmly				
Intercept	2.642	0.301	8.79	0.0000
Chlorophyll-*a*	0.107	0.065	1.63	0.1172
Zooplankton density	-0.072	0.037	-1.93	0.0659
**Phytoplankton richness**	**0.008**	**0.004**	**2.23**	**0.0359**
Macrophyte richness	0.001	0.005	0.25	0.8079
Environment (PCA-Axis 1)	-0.020	0.107	-0.18	0.8549
Hydrology	0.051	0.096	0.53	0.5995

## Discussion

Our results did not support the expectation that we would find models with higher coefficients of determination for the richness of algal groups than for the total species richness. However, we found that aquatic macrophyte species richness was, in general, one of the main predictors of periphyton species richness.

There are at least three non-exclusive explanations for the lack of support for our first hypothesis. First, habitat heterogeneity (see discussion below) may create available niches for different functional groups, which would result in a strong general richness pattern for the global community (which is contrary to our expectation). Second, after we separated the whole data set into trait groups, we may have ended up with less variation in species richness, but with similar amounts of variation in the predictors, reducing the coefficient of variation of the models. Third, we cannot rule out the role of more specific processes shaping richness patterns of different algal groups, which were not included or not well represented by the predictor variables we used in our analyses. This would make the coefficients of determination lower as the only variation captured by the predictors would be the one already represented in the global community model. The use of the deconstructive approach [20] was important, however, to suggest relationships that were not detected when using total species richness as the response variable (see the relationship between species richness of small/mobile algae and phytoplankton species richness and between species richness of large/mobile algae and hydrology).

We are aware that our results may be not extrapolated to other seasons (due to the likely seasonal dynamics of the periphytic community) and about the difficulties in establishing cause and effect relationships in observational studies [53]. However, our results support the hypothesis that periphyton species richness correlates positively with environmental heterogeneity (as proxied by macrophyte species richness; see [45]). Macrophyte species richness may contribute to environmental heterogeneity [54, 55], leading to increased periphytic species richness. Although this study was carried out only with the periphytic community associated with *E*. *azurea*, it is likely that variation in macrophyte species richness would be related to the creation of different substrate types and environmental conditions required by different periphytic species. In this case, different environmental conditions increase the colonisation opportunities for the periphytic matrix within the same area. A recent meta-analysis by Stein et al. [45] has also revealed a strong association between environmental heterogeneity (particularly promoted by plant diversity) and species richness of terrestrial plants and animals. Considering our results, aquatic macrophytes can promote increased environmental heterogeneity at different spatial scales due to their diversity in terms of architecture and texture [11, 56] and potentially facilitating the coexistence of different periphytic algal species.

Phytoplankton species richness was also a significant predictor of small/mobile and large/firmly attached algae species richness. This positive relationship may be partially explained by the exchange of algal cells between the periphytic communities and those suspended in the water column [57–59]. As indicated above (see *Planktonic and aquatic macrophyte communities* section), periphytic species are common in plankton samples [41; 60]; therefore, one can predict that environments with higher plankton species richness contribute with more colonists to the periphytic matrix [60]. Assuming that at least part of the variation in periphyton species richness is mediated by the variation in the richness of potential colonists (from other macrophytes and the planktonic environment), these results indicate the importance of explanatory variables obtained at scales larger than that at which the periphyton is sampled (i.e., micro-scale). Positive relationships between periphyton species richness and phytoplankton species richness may suggest, however, that both groups respond similarly to environmental conditions. To account for this possibility, we regressed species richness of small/mobile and of large/firmly attached on all explanatory variables, except phytoplankton species richness and correlated the residuals of these models with the residuals obtained after regressing phytoplankton species richness on all explanatory variables. The correlation coefficients were statistically significant (*r* = 0.37, *P* = 0.042 for small/mobile and *r* = 0.42, *P* = 0.020 for large/firmly). Thus, we think that the hypothesis of similar response to environmental conditions is unlikely to explain our results. However, we cannot discard the possibility that both periphyton and phytoplankton propagules are passive and similarly dispersed by water flow, generating a correlation between species richness of both groups, even if phytoplankton is not an important source of species for the periphyton community.

The relationship between species richness and productivity (usually proxied by chlorophyll-*a* concentration; see results for small/mobile algae) has been widely investigated in the ecological literature [61, 62], with some authors reporting linear (positive) relationships, quadratic relationships and even lack of such relationships [62]. At a local scale, this positive relationship may be interpreted in light of the “more individuals hypothesis” [63]. The positive relationship between species richness and productivity may be explained by a lower rate of local extinction in more productive environments, which usually exhibit higher population size and higher variation in species composition among sites [64]. Indeed, Chase [64] showed that more productive environments harbour a higher portion of the regional species pool compared to less productive ones. Considering that the sampling area was standardised in the present study, these ecological explanations seem more plausible than explanations based on sampling and statistical artefacts [45, 62].

We found that species richness of the whole community and large/loosely-attached algae was negatively related with the first principal component axis summarizing our abiotic data. This axis was negatively correlated with total phosphorus concentration and, to a lesser degree, with water temperature. These results suggest a positive association between species richness and total phosphorus, which is also consistent with the hypothesis that productivity is a key factor accounting for variation in species richness. We expected to find positive relationships between the richness of algal groups and ionic concentrations. However, this prediction was not supported by the results. Indeed, note that PC1 was positively correlated with conductivity and inorganic nitrogen content and, therefore, a negative relationship between these variables and species richness emerged. One possible explanation for this pattern is that TSM was also positively correlated with PC1 and, consequently negatively correlated with species richness. Thus, light limitation may be an important factor accounting for periphytic species richness [8, 65], even considering a relatively high ionic concentration. In general, these results highlight the role species sorting mechanisms in structuring periphyton community structure in the Upper Paraná River floodplain, both in terms of species richness (this study) and community composition [34].

We did not find a significant relationship between zooplankton density and periphyton species richness. In general, the role of grazing on periphyton species richness may be complex and dependent on several factors, including nutrient concentrations, light and type of ecosystems [7, 8]. More importantly, our results suggest that the density of herbivorous zooplankters, even considering that they can feed on organisms dislodged from macrophyte surfaces, is a poor proxy for grazing pressure on periphyton.

Our results did not support the hypothesis of increased coefficients of determination for deconstructed groups. Despite the inclusion of different environmental and biotic variables in this study, we cannot disregard the effect of other variables not measured, as well as combined effects of the interactions between distinct variables (e.g., light, nutrients and grazing, [8]) in shaping the richness of periphytic algae. However, the deconstructive approach was useful to suggest the importance of habitat heterogeneity (associated to aquatic macrophyte richness), environmental factors (mainly total P and TSM) and the size of the species pool as drivers of local species richness, independently of the high morphological variability of the species comprising the periphytic matrix.

## Supporting information

S1 Table Sub-basins, geographic coordinates and hydrology of the sampling environments in the Upper Paraná river floodplain. (DOCX)

S2 Table Periphytic algal species within groups formed based on attachment strategies and size. (DOCX)

S1 File List of references used to identify zooplankton species. (DOCX)

S2 File Description of the best models. (DOCX)
