# Supplementary material for: Main predictors of periphyton species richness depend on adherence strategy and cell size
Source: PLoS One. 2017 Jul 24;12(7):e0181720. doi: 10.1371/journal.pone.0181720 (PMC5524394; doi:10.1371/journal.pone.0181720)
Supplement: S1 Table — (DOCX) [file pone.0181720.s001.docx]

S1 Table. Sub-basins, geographic coordinates and hydrology of the sampling environments in the Upper Paraná river floodplain.

| Environments | Sub-basins | Geographic Coordinates | Hidrology |
| --- | --- | --- | --- |
| Peroba lake | Ivinhema | 22º54'30.3"S; 53º38'24.3"W | Lentic |
| Ventura lake | Ivinhema | 22º51'23.7"S; 53º36'1.02"W | Lentic |
| Lagoa Zé Paco lake | Ivinhema | 22º50'3.72"S; 53º34'18"W | Lentic |
| Ipoitã channel | Ivinhema | 22º50'7.56"S; 53º33'43.5"W | Lotic |
| Boca Ipoitã lake | Ivinhema | 22º50'7.92"S; 53º33'55.38"W | Lentic |
| Patos lake | Ivinhema | 22º49'33.66"S; 53º33'9.9"W | Lentic |
| Capivara lake | Ivinhema | 22º47'56.52"S; 53º32'5.4"W | Lentic |
| Ivinhema river | Ivinhema | 22º47'59.64"S; 53º32'21.3"W | Lotic |
| Finado Raimundo lake | Ivinhema | 22º47'57.6"S; 53º32'29.16"W | Lentic |
| Jacaré lake | Ivinhema | 22º47'2.04"S; 53º29'49.08"W | Lentic |
| Sumida lake | Ivinhema | 22º46'54.78"S; 53º29'22.2"W | Lentic |
| Cervo lake | Ivinhema | 22º46'29.58"S; 53º29'46.98"W | Lentic |
| Curutuba channel | Baía | 22º45'2.88"S; 53º21'32.22"W | Lotic |
| Traíra lake | Baía | 22º44'45.6"S; 53º20'21.66"W | Lentic |
| Guaraná lake | Baía | 22º43'16.68"S; 53º18'9.24"W | Lentic |
| Baía river | Baía | 22º43'23.16"S; 53º17'25.5"W | Lotic |
| Fechada lake | Baía | 22º42'37.92"S; 53º16'33.06"W | Lentic |
| Pousada das Garças lake | Baía | 22º42'1.14"S; 53º15'23.52"W | Lentic |
| Porcos lake | Baía | 22º42'4.44"S; 53º14'40.08"W | Lentic |
| Aurélio lake | Baía | 22º41'34.68"S; 53º13'50.58"W | Lentic |
| Baía channel | Baía | 22º41'26.94"S; 53º13'29.34"W | Lotic |
| Maria Luiza lake | Baía | 22º40'30.18"S; 53º13'11.16"W | Lentic |
| Gavião lake | Baía | 22º40'47.94"S; 53º13'53.46"W | Lentic |
| Onça lake | Baía | 22º39'48.42"S; 53º12'1.62"W | Lentic |
| Bilé lake | Paraná | 22º45'13.56"S; 53º17'9.48"W | Lentic |
| Leopoldo lake | Paraná | 22º45'24"S; 53º16'7.98"W | Lentic |
| Pau Véio lake | Paraná | 22º44'50.76"S; 53º15'11.16"W | Lentic |
| Garças lake | Paraná | 22º43'27.18"S; 53º13'4.56"W | Lentic |
| Pombas lake | Paraná | 22º47'55.92"S; 53º21'32.58"W | Lentic |
| Cortado channel | Paraná | 22º48'43.44"S; 53º22'40.92"W | Lotic |
