# Supplementary material for: Main predictors of periphyton species richness depend on adherence strategy and cell size
Source: PLoS One. 2017 Jul 24;12(7):e0181720. doi: 10.1371/journal.pone.0181720 (PMC5524394; doi:10.1371/journal.pone.0181720)
Supplement: S2 Table — (DOCX) [file pone.0181720.s002.docx]

S2 Table. Periphytic algal species within groups formed based on attachment strategies and size.

| **Mobile (< 70 µm)** |  |
| --- | --- |
| *Chlamydomonas* sp. | *Trachelomonas* *volvocina* (Ehrenberg) Ehrenberg |
| *Cryptomonas* sp. | *Synura* sp. |
| *Gonium pectorale* Müller | *Frustulia rhomboides* (Ehrenberg) De Toni |
| *Euglena* sp. 1 | *Gyrosigma acuminatum* (Kützing) Rabenhorst |
| *Phacus* *contortus* Bourrelly | *Navicula capitatoradiata* Germain |
| *Phacus* *curvicauda* Svirenko | *Navicula* cf *constans* Hustedt |
| *Phacus* *orbicularis* Hübner | *Navicula cryptocephala* Kützing |
| *Phacus* *suecius* Lemmermann | *Navicula schroeteri* Meister |
| *Phacus* sp. 1 | *Navicula viridula* (Kützing) Ehrenberg |
| *Phacus* sp. 2 | *Navicula* sp. 1 |
| *Phacus* sp. 3 | *Navicula* sp. 2 |
| *Strombomonas* cf *fluviatilis* (Lemmermann) Deflandre | *Nitzschia amphibia* Grunow |
| *Trachelomonas* cf *abrupta* Svirenko | *Nitzschia* cf *linearis* Smith |
| *Trachelomonas armata* (Ehrenberg) Stein | *Nitzschia palea* (Kützing) Smith |
| *Trachelomonas* *hispida* (Perty) Stein | *Pinnularia acrosphaeria* Smith |
| *Trachelomonas* cf *oblonga* Lemmermann | *Pinnularia* cf *braunii* (Grunow) Cleve |
| *Trachelomonas* *sculpta* Balech | *Pinnularia divergens* Smith |
| *Trachelomonas* *verrucosa* Stokes | *Pinnularia* cf *mesolepta* (Ehrenberg) Smith |
| **Mobile (> 70 µm)** |  |
| *Euglena acus* (Müller) Ehrenberg | *Nitzschia acicularis* (Kützing) Smith |
| *Euglena* sp. 2 | *Nitzschia* cf *lorenziana* Grunow |
| *Euglena* sp. 3 | *Pinnularia gibba* Ehrenberg |
| *Phacus undulatus* (Skvortzov) Pochmann | *Pinnularia* sp. |
| *Mallomonas* sp. | *Surirela linearis* Smith |
| *Gyrosigma* sp. |  |
| **Loosely attached (< 70 µm)** |  |
| *Ankistrodesmus densus* Korshikov | *Cosmarium* sp. 1 |
| *Ankistrodesmus fusiformis* Corda | *Cosmarium* sp. 2 |
| *Coelastrum cambricum* Archer | *Cosmarium* sp. 3 |
| *Crucigenia fenestrada* (Schmidle) Schmidle | *Cosmarium* sp. 4 |
| *Crucigenia quadrata* Morren | *Cosmarium* sp. 5 |
| *Crucigeniella* sp. | *Cosmarium* sp. 6 |
| *Desmodesmus brasiliensis* (Bohlin) Hegewald | *Cosmarium* sp. 7 |
| *Desmodesmus quadricauda* (Turpin) ? | *Cosmarium* sp. 8 |
| *Dimorphococcus lunatus* Braun | *Cosmarium* sp. 9 |
| *Elakatothrix* sp. | *Cosmarium* sp. 10 |
| *Kirchneriella* cf *aperta* Teiling | *Cosmarium* sp. 11 |
| *kirchneriella lunaris* (Kirchner) Möbius | *Cosmocladium* sp. |
| *Kirchneriella obesa* (West) West & West | *Euastrum abruptum* Reinsch |
| *Monoraphidium arcuatum* (Korshikov) Hindák | *Euastrum bidentatum* Nägeli |
| *Monoraphidium convolutum* (Corda) Komárková-Legnerová | *Euastrum binale* Ehrenberg & Ralfs |
| *Monoraphidium griffithi* (Berkeley) Komárková-Legnerová | *Euastrum denticulatum* Gay |
| *Monoraphidium tortile* (West & West) Komárková-Legnerová | *Euastrum elegans* (Brébisson) Kützing & Ralfs |
| *Nephrocytium agardhianum* Nägeli | *Euastrum* cf *erosum* Lundell |
| *Nephrocytium lunatum* West | *Euastrum gemmatum* Ralfs |
| *Oocystis lacustris* Chodat | *Euastrum rectangulare* Fritsch & Rich |
| *Pediastrum duplex* Meyen | *Euastrum* cf *subintegrum* Nordstedt |
| *Radiococcus* sp. | *Euastrum sublobatum* Brébisson & Ralfs |
| *Scenedesmus acuminatus* (Lagerheim) Chodat | *Euastrum* cf *validum* West & West |
| *Scenedesmus alternans* Reinsch | *Euastrum* sp. 1 |
| *Scenedesmus arcuatus* (Lemmermann) Lemmermann | *Euastrum* sp. 2 |
| *Scenedesmus bijugus* (Turpin) Lagerheim | *Euastrum* sp. 3 |
| *Scenedesmus brevispina* (Smith) Chodat | *Octacanthidium mucronulatus* Nordstedt |
| *Scenedesmus denticulatus* (Lagerheim) An, Friedl & Hegewald | *Staurastrum brevispina* Brébisson |
| *Scenedesmus* cf *hystrix* (Lagerheim) Hegewald | *Staurastrum* cf *disputatum* West & West |
| *Scenedesmus* cf *parisiensis* Chodat | *Staurastrum* cf *iotanum* Wolle |
| *Scenedesmus* sp. | *Staurastrum irregulare* West & West |
| *Schroederia antillarum* Komárek | *Staurastrum margaritaceum* Meneghini & Ralfs |
| *Selenastrum bibraianum* Reinsch | *Staurastrum muticum* Brébisson & Ralfs |
| *Selenastrum gracile* Reinsch | *Staurastrum quadrangulare* Brébisson & Ralfs |
| *Selenastrum rinoi* Komárek & Comas | *Staurastrum* cf *rotula* Nordstedt |
| *Selenastrum* sp. | *Staurastrum* cf *sebaldi* Reinsch |
| *Tetraedron caudatum* (Corda) Hansgirg | *Staurastrum teliferum* Ralfs |
| *Tetraedron minimum* (Braun) Hansgirg | *Staurastrum* cf *tetracerum* Ralfs & Ralfs |
| *Tetraedron regulare* Kützing | *Staurastrum* cf *trifidum* Nordstedt |
| *Tetraedron trigonum* (Nägeli) Hansgirg | *Staurastrum zonatum* Børgesen |
| *Tetrastrum komarekii* Hindák | *Staurastrum* sp. 1 |
| *Tetrastrum mitrae* (Tiwari & Pandey) Komárek | *Staurastrum* sp. 2 |
| *Tetrastrum* sp. | *Staurastrum* sp. 3 |
| Chlorophyceae 1 | *Staurastrum* sp. 4 |
| Chlorophyceae 2 | *Staurastrum* sp. 5 |
| Chlorophyceae 3 | *Staurastrum* sp. 6 |
| *Actinotaenium* cf *globosum* (Bulnheim) Förster & Còmpere | *Staurastrum* sp. 7  *Staurodesmus cuspidatus* (Brébisson) Teiling |
| *Arthrodesmus* sp. | *Staurodesmus* cf *dejectus* (Brébisson) Teiling |
| *Closterium incurvum* Brébisson | *Staurodesmus* cf *dickiei* (Ralfs) Lillieroth |
| *Closterium navicula* (Brébisson) Lütkemülle | *Staurodesmus triangularis* (Lagerheim) Teiling |
| *Closterium* sp. 1 | *Staurodesmus* cf *validus* (West & West) Thomasson |
| *Cosmarium abbreviatum* (Bulnheim) Förster ex Compère | *Staurodesmus* sp. 1 |
| *Cosmaruim* cf *bioculatum* Brébisson | *Staurodesmus* sp. 2 |
| *Cosmarium bireme* Nordstedt | *Staurodesmus* sp. 3 |
| *Cosmarium blyttii* Wille | *Staurodesmus* sp. 4 |
| *Cosmarium* cf *brebissonii* Meneghini ex Ralfs | *Staurodesmus* sp. 5 |
| *Cosmarium candianum* Delponte | *Staurodesmus* sp. 6 |
| *Cosmarium comissurale* Brébisson & Ralfs | *Tetraplektron torsum* (Turner) Dedusenko-Shchegoleva |
| *Cosmarium* cf *contractum* Kirchner | Xanthophyceae 1 |
| *Cosmarium* cf *depressum* (Nägeli) Lundell | Xanthophyceae 2 |
| *Cosmarium excavatum* Nordstedt | *Borzia* sp. |
| *Cosmarium granatum* Brébisson ex Ralfs | *Chroococcus* cf *dispersus* (Keissler) Lemmermann |
| *Cosmarium* cf *hexagonum* Nordstedt | *Chroococcus* sp. |
| *Cosmarium humile* (Gay) Nordstedt | *Coelomoron* cf *tropicale* Senna, Peres & Komárek |
| *Cosmarium impressulum* Elfving | *Gloeocapsa* cf. *fuscolutea* Nägeli & Kützing |
| *Cosmarium lagoense* Nordstedt | *Merismopedia tenuissima* Lemmermann |
| *Cosmarium leave* Rabenhorst | *Pseudanabaena* cf *frigida* (Fritsch) Anagnostidis |
| *Cosmarium* cf *ornatum* Ralfs ex Ralfs | *Pseudanabaena moliniformes* Komárek & Kling |
| *Cosmarium* cf *phaseolus* Brébisson ex Ralfs | *Pseudanabaena* sp. 1 |
| *Cosmarium pseudoprotuberans* Kirchner | *Pseudanabaena* sp. 2 |
| *Cosmarium* cf *pyramidatum* Brébisson ex Ralfs | *Synecocystes* sp. |
| *Cosmarium rectangulare* Grunow | Cyanobacteria 3 |
| *Cosmarium regnellii* Wille | Dinophyceae 1 |
| *Cosmarium regnesi* Reinsch | *Cyclotella* cf *meneghiniana* Kützing |
| *Cosmarium reniforme* (Ralfs) Archer | *Diplonies subovalis* Cleve |
| *Cosmarium* cf *subnudiceps* West & West | *Melosira varians* Agardh |
| *Cosmarium trilobulatum* Reinsch | *Staurosira elliptica* (Schumann) Williams & Round |
| *Cosmarium vexatum* West |  |
| **Loosely attached (> 70 µm)** |  |
| *Ankistrodesmus falcatus* (Corda) Ralfs | *Pleurotaenium* cf *ehrenbergii* (Ralfs) Delponte |
| *Closteriopsis acicularis* (Smith) Belcher & Swale | *Pleurotaenium* cf *nodosum* (Bailey & Ralfs) Lundell |
| *Closteriopsis* sp. | *Pleurotaenium trabecula* Nägeli |
| *Coelastrum astroideum* De Notaris | *Spondylosium* cf *pulchellum* (Archer) Archer |
| *Coelastrum microporum* Nägeli | *Spirogyra* sp. 1 |
| *Dictyosphaerium erhenbergianum* Nägeli | *Spirogyra* sp. 2 |
| *Dictyosphaerium pulchellum* Wood | *Staurastrum leptacanthum* Nordestedt |
| *Glaucocystis* sp. | *Staurastrum leptocladum* Nordstedt |
| *Gloeocystis* sp. | *Staurastrum minesotense* Wolle |
| *Monoraphidium* sp. | *Staurastrum setigerum* Cleve |
| *Oonephris obesa* (West & West) Fott | *Teilingia excavata* (Ralfs & Ralfs) Bourrelly |
| *Pediastrum tetras* (Ehrenberg) Ralfs | *Teilingia granulata* (Roy & Bisset) Bourrelly |
| *Selenodiction brasiliense* | *Xanthidium trilobum* Nordstedt |
| *Sorastrum spinulosum* Nägeli | *Xanthidium* sp. 1 |
| *Closterium acutum* Brébisson | *Xanthidium* sp. 2 |
| *Closterium calosporum* Wittrock | *Ophiocytium cochleare* (Eichwald) Braun |
| *Closterium closterioides* (Ralfs) Louis & Peeters | *Ophiocytium parvulum* (Perty) Braun |
| *Closterium cornu* Ehrenberg & Ralfs | *Ophiocytium* sp. 1 |
| *Closterium dianae* Ehrenberg & Ralfs | *Ophiocytium* sp. 2 |
| *Closterium jenneri* Ralfs | *Anabaena constricta* (Szafer) Geitler |
| *Closterium lineatum* Ehrenberg & Ralfs | *Anabaena* sp. 1 |
| *Closterium moniliferum* Ehrenberg & Ralfs | *Anabaena* sp. 2 |
| *Closterium pusillum* Hantzsch | *Anabaena* sp. 3 |
| *Closterium setaceum* Ehrenberg & Ralfs | *Anabaena* sp. 4 |
| *Closterium tortum* Griffiths | *Aphanocapsa hyalina* (Lyngbye) Hansgirg |
| *Closterium toxon* West | *Aphanocapsa grevillei* (Berkeley) Rabenhorst |
| *Closterium venus* Kützing & Ralfs | *Geitlerinema splendidum* (Greville & Gomont) Anagnostidis |
| *Closterium* sp. 2 | *Jaaginema quadripunctulatum* (Brühl *&* Bisw) Anagnostidis & Komárek |
| *Cosmarium conspersum* Ralfs | *Komvophoron schmidlei* (Jaag) Anagnostidis & Komárek |
| *Cosmarium margaritatum* (Lundell) Roy & Bisset | *Lyngbya* sp. |
| *Cosmarium panamense* Prescott | *Microcystis* sp. |
| *Cosmarium* sp. 12 | *Oscillatoria annae* Goor |
| *Cosmarium* sp. 13 | *Oscillatoria ornata* Kützing & Gomont |
| *Euastrum verrucosum* Ehrenberg & Ralfs | *Oscillatoria perornata* Skuja |
| *Gonatozygon brebissonii* De Bary | *Oscillatoria princeps* Vaucher & Gomon |
| *Gonatozygon monotaenium* De Bary | *Oscillatoria* sp. 1 |
| *Gonatozygon pilosum* Wolle | *Oscillatoria* sp. 2 |
| *Hyalotheca dissiliens* Brébisson & Ralfs | *Pseudanabaena limnetica* (Lemmermann) Komárek |
| *Micrasterias furcata* Agardh & Ralfs | *Phormidium* sp. |
| *Micrasterias laticeps* Nordstedt | Cyanobacteria 1 |
| *Micrasterias mahabuleshwarensis* Hobson | Cyanobacteria 2 |
| *Micrasterias truncata* Brébisson & Ralfs | *Aulacoseira ambigua* (Grunow) Simonsen |
| *Mougeotia* sp. | *Aulacoseira granulata* (Ehrenberg) Simonsen |
| *Onychonema leave* Nordstedt | *Amphipleura lindheimeri* Grunow |
| *Pleurotaenium* cf *cylindricum* (Turner) Schimidle |  |
| **Firmly attached (< 70 µm)** |  |
| *Aphanochaete repens* Braun | *Pseudanabaena mucicola* (Naumann & Huber-Pestalozzi) Schwabe |
| *Chaetosphaeridium globosum* (Nordstedt) Klebahn | *Xenococcus* sp. |
| *Characiellopsis skujae* (Fott) Komárek | *Achnanthes inflata* (Kützing) Grunow |
| *Characium conicum* Korshikov | *Achnanthidium minutissimum* (Kützing) Czarnecki |
| *Characium* *indicum* Patel & Isabella | *Amphora copulata* (Kützing) Schoeman & Archibald |
| *Characium guttula* Playfair | *Cymbella* sp. 1 |
| *Characium ornitocephalum* Braun | *Cymbella* sp. 2 |
| *Characium* sp. 1 | *Eunotia bilunaris* (Ehrenberg) Schaarschmidt |
| *Characium* sp. 2 | *Eunotia camelus* Ehrenberg |
| *Characium* sp. 3 | *Eunotia curvata* (Kützing) Lagerstedt |
| Chlorophyceae 1 | *Eunotia didyma* Grunow |
| *Characiopsis aquilonaris* Skuja | *Eunotia sudetica* Otto Müller |
| *Characiopsis longipes* (Braun) Borz | *Eunotia* sp. 1 |
| *Characiopsis minor* Pascher | *Eunotia* sp. 2 |
| *Characiopsis pyriformis* (Braun) Borzí | *Eunotia* sp. 3 |
| *Characiopsis* sp. | *Eunotia* sp. 4 |
| *Dioxys* sp. | *Encyonema mesianum* (Cholnoky) Mann |
| Xanthophyceae 3 | *Encyonema minutum* (Hilse) Mann |
| *Colacium* sp. | *Encyonema silesiacum* (Bleisch) Mann |
| *Stylodinium* sp. | *Fragilaria capucina* Desmazières |
| *Lagynion* sp. | *Gomphonema affine* Kützing |
| *Salpingoeca* sp. 1 | *Gomphonema apicatum* Ehrenberg |
| *Salpingoeca* sp. 2 | *Gomphonema augur* Ehrenberg |
| *Salpingoeca* sp. 3 | *Gomphonema gracile* Ehrenberg |
| Chrysophyceae 1 | *Gomphonema parvulum* Kützing |
| Chrysophyceae 2 | *Gomphonema pumilum* (Grunow) Reichardt & Lange-Bertalot |
| Chrysophyceae 3 | *Gomphonema subtile* Ehrenberg |
| Chrysophyceae 4 | *Gomphonema truncatum* Ehrenberg |
| *Calothrix brevissima* West | *Gomphonema* sp. 1 |
| *Calothrix* sp. | *Gomphonema* sp. 2 |
| *Chaemosiphon* sp. | *Gomphonema* sp. 3 |
| *Nostoc* sp. | *Synedra goulardii* Brébisson ex Cleve & Grunow |
| **Firmly attached (> 70 µm)** |  |
| *Chaetophora* sp. | *Leibleinia epiphytica* (Hieronymus) Còmpere |
| *Coleochaete irregularis* Pringsheim | *Rivularia* sp. |
| *Coleochaete orbicularis* Pringsheim | Cyanobacteria 4 |
| *Coleochaete* sp. | *Bulbochaete* sp. |
| *Stigeoclonium* sp. | *Oedogonium reinschii* Roy & Hirn |
| *Ulothrix* sp. | *Oedogonium* sp. 1 |
| *Uronema* sp. 1 | *Oedogonium* sp. 2 |
| *Uronema* sp. 2 | *Oedogonium* sp. 3 |
| *Audouinella* sp. | *Oedogonium* sp. 4 |
| *Compsopogon* sp. | *Dinobryum sertularia* Ehrenberg |
| *Calothrix fusca* Bornet & Flahault | *Eunotia flexuosa* (Brébisson & Kützing) Kützing |
| *Gloeothrichia* sp. | *Eunotia lineolata* Hustedt |
| *Hapalosiphon* sp. | *Eunotia zygodon* Ehrenberg |
| *Heteroleibleinia epiphytica* Komárek | *Eunotia* sp. 5 |
| *Heteroleibleinia kossinskajae* (Elenkin) Anagnostidis & Komárek | *Gomphonema turris* Ehrenberg |
| *Heteroleibleinia ucrainica* (Schirschoff) Anagnostidis & Komárek | *Gomphonema* sp. 4 |
| *Heteroleibleinia* sp. 1 | *Stenopterobia delicatissima* (Lewis) Brébisson & van Heurck |
| *Heteroleibleinia* sp. 2 | *Ulnaria ulna* (Nitzsch) Compère |
