# Supplementary material for: Main predictors of periphyton species richness depend on adherence strategy and cell size
Source: PLoS One. 2017 Jul 24;12(7):e0181720. doi: 10.1371/journal.pone.0181720 (PMC5524394; doi:10.1371/journal.pone.0181720)
Supplement: S1 File — (DOCX) [file pone.0181720.s003.docx]

**List of references used to identify zooplankton species**

Deflandre G. Le genre Arcella Ehrenberg. Archiv für Protistenkunde. 1928; 64: 152- 287.

Deflandre G. Le genre Centropyxis Stein. Archiv für Protistenkunde. 1929; 67: 322-375.

Elmoor-Loureiro MAL. Manual de identificação de cladóceros límnicos do Brasil. Brasília: Editora Universa; 1997. 155p.

Gauthier-Lièvre L, Thomas R. Le genre Difflugia, Pentagonia, Maghrebia et Hoogenraadia (Rhizopodes Testacès) en Afrique. Archiv für Protistenkunde. 1958; 103: 1-370.

Koste W. Rotatoria die Rädertiere Mitteleuropas begründet von Max Voight. Monogononta. Berlin: Gebrüder Borntraeger; 1978. 673 p.

Matsumura-Tundisi T. Latitudinal distribution of Calanoida copepods in freshwater aquatic systems of Brazil. Braz J Biol. 1986; 46: 527-553.

Reid JW. Chave de identificação e lista de referências bibliográficas para as espécies continentais sulamericanas de vida livre da ordem Cyclopoida (Crustacea, Copepoda). Boletim de Zoologia. 1985; 9: 17-143. doi: [10.11606/issn.2526-3358.bolzoo.1985.122293](http://dx.doi.org/10.11606/issn.2526-3358.bolzoo.1985.122293)

Segers H. Rotifera: the Lecanidae (Monogonta). Guides to the identification of the micro invertebrates of the continental water of the world. The Hague: SPB Academic; 1995. 226 pp.
