# Supplementary material for: Main predictors of periphyton species richness depend on adherence strategy and cell size
Source: PLoS One. 2017 Jul 24;12(7):e0181720. doi: 10.1371/journal.pone.0181720 (PMC5524394; doi:10.1371/journal.pone.0181720)
Supplement: S2 File — (DOCX) [file pone.0181720.s004.docx]

**Description of the best models**

The best model for total species richness (with an AIC of 37.5) was the one without spatial error structure. The models allowing for spatial structure in the residuals were (with a mean AIC of 41.52), on average, 4 AIC units apart from the best-supported model and, therefore, they are considerably less plausible to describe the data. Similarly, the best GLS models for small/loosely attached (AIC = 63.25), large/loosely attached (AIC = 58.63), small/firmly attached (AIC = 19.87), and large/firmly attached (AIC = 33.12) were the ones without spatial error structure; on average, AIC values for those models allowing for spatial structure in the residuals were, on average, equal to 67.25, 62.63, 23.45 and 35.35 respectively. However, the best GLS models for small (AIC = 36.48) and large (AIC = 74.88) mobile species were the ones with spatial structure in the residuals (rational quadratic correlation). The AIC values for the other models were, on average, equal to 40.16 and 82.70.
